# Research frontiers and hotspots in optical coherence tomography applications among patients with acute coronary syndromes: Bibliometric and visual analysis

**DOI:** 10.1097/MD.0000000000040216

**Published:** 2024-10-25

**Authors:** BuChun Zhang, Yi Zhang, Hui Li, Nan Chen

**Affiliations:** a Department of Cardiology, The First Affiliated Hospital of USTC, Division of Life Sciences and Medicine, University of Science and Technology of China, Anhui Hefei, China; b Graduate School, Wannan Medical College, Anhui Wuhu, China.

**Keywords:** acute coronary syndrome, bibliometrics, optical coherence tomography

## Abstract

Research on the use of optical coherence tomography (OCT) in acute coronary syndrome (ACS) has increased in recent years. However, a comprehensive analysis of the trends and hotspots in OCT research is currently lacking. The objective of this study was to identify global trends in research on OCT in ACS from a bibliometric perspective and to provide researchers with new research hotspots. Relevant literature from 1998 to 2023 was retrieved from the Web of Science Core Collection. CiteSpace and VOSviewer software were used to collect and analyze publication trends in related fields. A total of 965 publications from 58 countries and 1389 institutions were included in the present study. We found that Japan produced the most publications (20.83%, 201), followed by the United States (19.90%, 192), and China (14.09%, 136). However, the United States has the highest total number of citations in this field. Harvard Medical School and Harbin Medical University had the highest numbers of publications and citations. The journal with the most publications was the International Journal of Cardiology. Plaque erosion, calcified nodules, and intracoronary imaging are the most recent research hotspots and frontiers. Our work summarizes 25 years of OCT research in the ACS, highlighting hotspots, key themes, and emerging frontiers to help guide future research.

## 1. Introduction

Optical Coherence Tomography (OCT) is a high-resolution intravascular imaging modality that can reveal a more detailed coronary plaque morphology than other intravascular imaging modalities.^[1]^ In 2009, the world’s first report on the in vivo imaging of coronary arteries using OCT was published.^[2]^ Since then, OCT has been progressively and rapidly developed in clinical research.

Acute Coronary Syndrome (ACS) is a leading cause of death worldwide. OCT has obvious advantages in identifying pathogenesis, clarifying diagnosis, and guiding treatment.^[3]^ Many researchers have conducted studies on the application of OCT in ACS. The use of OCT has changed pharmacological and percutaneous coronary intervention strategies for patients with ACS. However, the research themes and trends in this area are not yet clear. Currently, no studies have analyzed the current status and hotspots of research regarding the application of OCT in ACS in a bibliometric format.

Bibliometrics is a set of methods used to quantitatively analyze academic literature in terms of number, citations, and relevance metrics in a particular field of research.^[4]^ It allows researchers to understand the structure of knowledge and to explore trends in research. In this study, we conducted a comprehensive analysis of research related to the application of OCT in ACS using bibliometric methods to summarize the state of research in this area. This study also analyzed research hotspots and emerging frontiers as a reference for future research directions.

## 2. Methods

### 2.1. Data source and collection

The literature on the use of intravascular OCT in patients with ACS was extracted from the Web of Science (WoS) Core Collection database, published between 1998 and 2023. Document type was restricted to articles and reviews, and the language was restricted to English. A total of 1259 publications remained after limiting the publication years from 1998 to 2023. The search formula used in this study was TS (subject heading) = optical coherence tomography OR OCT tomography AND TS=(“OCT” OR “coherence tomography” OR “optical” OR “tomography OCT”). AND((((((((((((((TS =(“ Acute Coronary Syndrome *” OR “Acute Coronary Syndrome*” OR “Syndrome* Acute Coronary”). The search results were exported as a plain text file, and the recorded content was “Full Record and Cited References” and stored in download _*. txt format. After screening, 965 records were included and no duplicates were found. The search was completed on January 02, 2024, to avoid data bias due to database updates.

The data used in this study were taken from public databases. Therefore, ethical approval was not required.

### 2.2. Data analysis and visualization

All records retrieved from WoS Core Collection database were independently downloaded by 2 authors. The information extracted from each study included title, authors, keywords, institutions, countries and regions, total citations, year of publication, journal, and impact factor. VOSviewer (Leiden University, Leiden, the Netherlands) and CiteSpace V (Drexel University, Philadelphia) were used for data analysis and visualization.

## 3. Results

### 3.1. Publication trends

**Figure 1.**
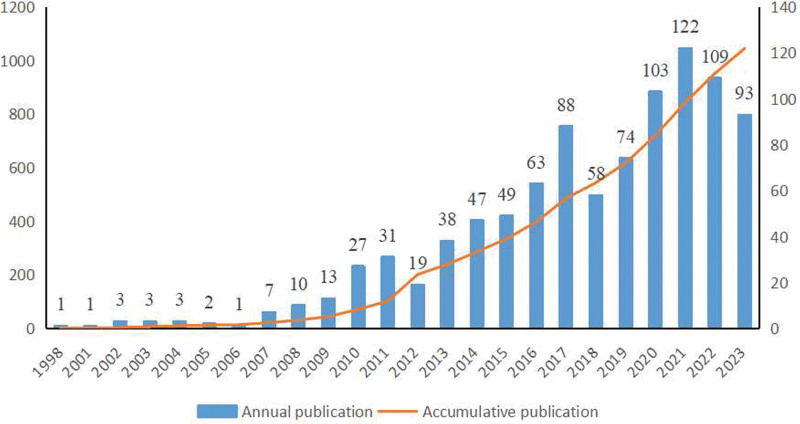
The cumulative and annual number of publications from 1998-2023.

A total of 1295 publications on the use of OCT in patients with ACS were identified in the WoS database from 1998 to 2023. Based on the selection criteria, 785 articles were identified and the remaining 180 were categorized as reviews. Figure 1 shows the annual and cumulative numbers of publications related to clinical research on OCT in patients with ACS. The cumulative number of publications increased steadily, from one publication in 1998 to 93 in 2013. By January 2024, the total number of citations had been 31,591. After excluding self-citations, 27,465 articles remained, with an average number of citations of 32.74 and an average H-index of 73. These data suggest that OCT is attracting increasing interest from researchers worldwide.

### 3.2. Analysis of the cooperative relationship

**Figure 2.**
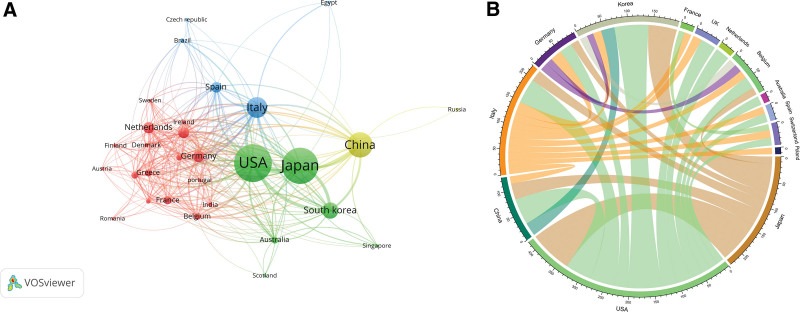
Analysis of countries related to research on the application of optical coherence tomography (OCT) in acute coronary syndrome (ACS). (A) Visual mapping of collaborative relationships between countries of related publications in VOSviewer. (B) A circle diagram evaluating international collaboration between countries.

**Figure 3.**
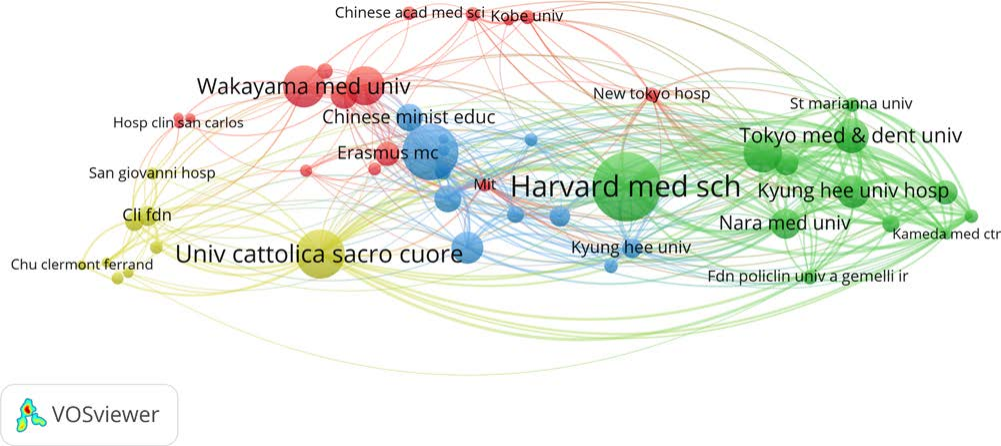
Visual mapping of collaborative relationships between related publishing institutions in VOSviewer.

The countries/regions, institutions, and authors included in the literature were visually analyzed using VOSviewer software. Each node in the map represents a country/region, institution, or author; the larger the node, the greater the influence. The distance and strength of the links between nodes indicate the degree of cooperation between the 2 points. From 1998 to 2023, 58 countries and 1389 institutions were involved in publishing OCT-related ACS research papers. Figure 2A illustrates national collaboration networks. Japan ranks first with 201 publications, followed by the USA (192 publications), and China (136 publications). According to our citation analysis, the USA (16,150 citations) was the most cited country, followed by Japan (7542 citations), and China (3525 citations). Figure 2B shows active collaboration between countries. The closest collaboration is between the USA and Japan, followed by Japan, which collaborates closely with China and South Korea. Figure 3 shows the network of institutional collaborations. Harvard Medical School in the USA published 84 papers, followed by Harbin Medical University in China with 67 papers. The top 10 most-cited institutions are from the USA with 6, followed by Japan with 2 (Table 1), indicating that these institutions have a significant position in the field of OCT.

**Table 1:** The top 10 research institutions with publications and citations.

Rank	Institution	Publications	Country	Institution	Citations	Country
1	Harvard Medical School	84	USA	Harvard University	4289	USA
2	Harbin Medical University	67	China	Harvard Medical School	2868	USA
3	Università Cattolica del Sacro Cuor	57	Italy	Wakayama Medical University	2316	Japan
4	Wakayama Medical University	48	Japan	Harbin Medical University	2147	China
5	Cardiovasc Res Fdn	45	USA	Università Cattolica del Sacro Cuor	2021	Italy
6	Tsuchiura Kyodo General Hospital	44	Japan	Cardiovasc Res Fdn	1754	USA
7	Tokyo Medical and Dental University	39	Japan	Columbia University	1699	USA
8	Kyung Hee University	37	South Korea	CVPath Institute	1578	USA
9	Harvard University	36	USA	Tsuchiura Kyodo General Hospital	1445	Japan
10	Nara Medical University	33	Japan	Massachusetts General Hospital	1430	USA

**Figure 4.**
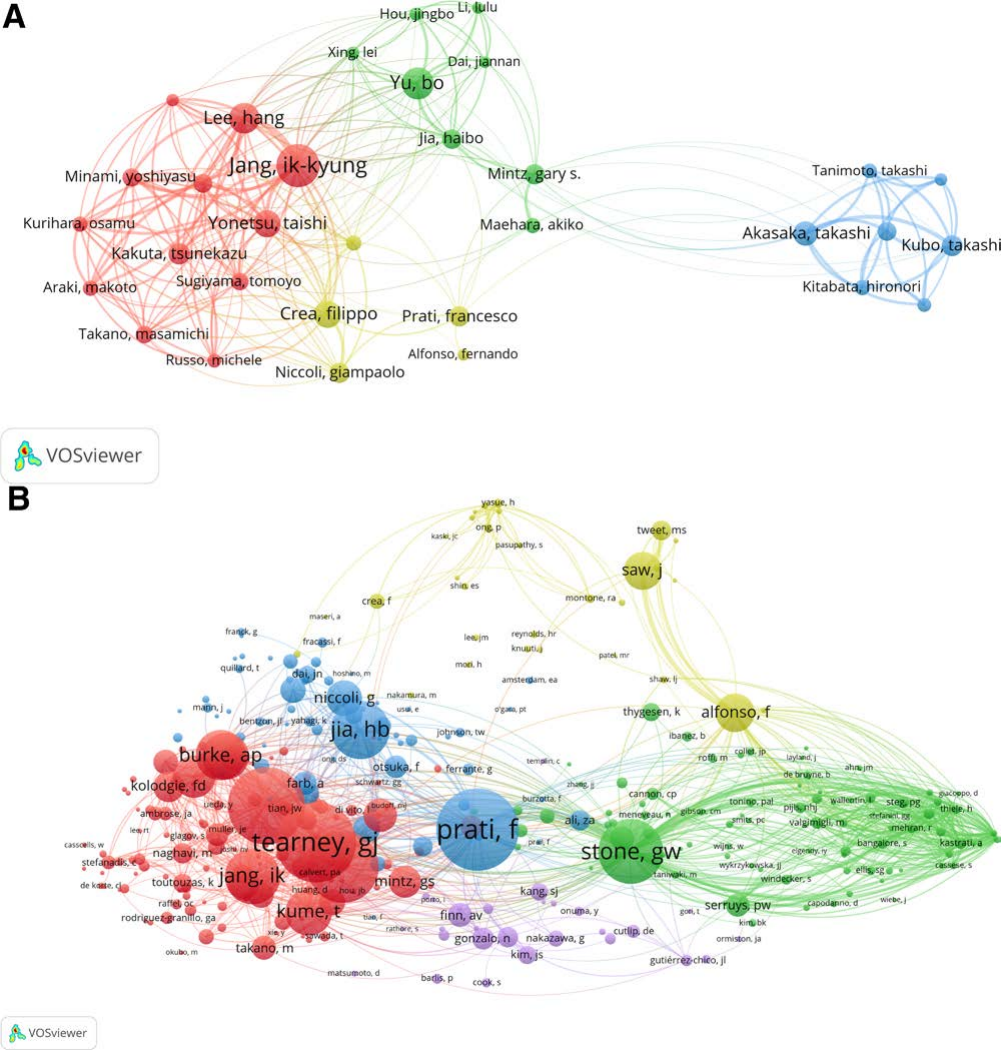
Network visualization map of co-authorship in optical coherence tomography (OCT) for acute coronary syndrome (ACS) publications in VOSviewer. (A) Visual map of co-authorship analysis. (B) Visual map of coauthorship analysis. OCT = optical coherence tomography.

The 10 most productive authors with the highest number of publications and citations on OCT applications in ACS are listed in Table 2. Figure 4 shows the authors’ co-authorship and cocitation maps. The collaborations of the authors’ network analysis showed that Jang Ik-Kyung, Crea Filippo, Yu Bo, and Akasaka Takashi were at the center of the collaborative network (Fig. 4A). Figure 4B shows the collaborative network among authors who have published more than 20 articles. These authors can be grouped into 5 main clusters, represented by 5 different colors. Each cluster represents a collaborative team and the figure shows the core authors within each cluster. Most authors who collaborated closely came from different institutions and collaborated more with foreign researchers. The citation network analysis classified the authors into 5 clusters: Stone GW, Serruys PW, etc. (green); Tearney GJ, Jang Ik-Kyung, etc. (red); Yu Bo, Jia Hb, etc. (blue); Saw J, Alfonso F, et al (yellow); Kang SJ, et al (purple) (Fig. 4B).

**Table 2:** The top 10 productive authors with publications and citation frequency.

Rank	Author	Publications	Country	Author	Citations	Country
1	Jang Ik-Kyung	80	USA	Jang Ik-Kyung	3012	USA
2	Yu Bo	58	China	Virmani Renu	2571	USA
3	Lee Hang	55	USA	Akasaka Takashi	2305	Japan
4	Crea Filippo	49	Italy	Narula Jagat	2079	USA
5	Akasaka Takashi	43	Japan	Crea Filippo	1867	Italy
6	Kakuta Tsunekazu	38	USA	Tanaka Atsushi	1756	Japan
7	Kubo Takashi	37	Japan	Lee Hang	1745	USA
8	Prati Francesco	36	USA	Kubo Takashi	1713	Japan
9	Mintz GS	35	USA	Kitabata Hironori	1587	Japan
10	Jia Haibo	34	China	Tanimoto Takashi	1570	Japan

### 3.3. Analysis of the journal distribution

**Figure 5.**
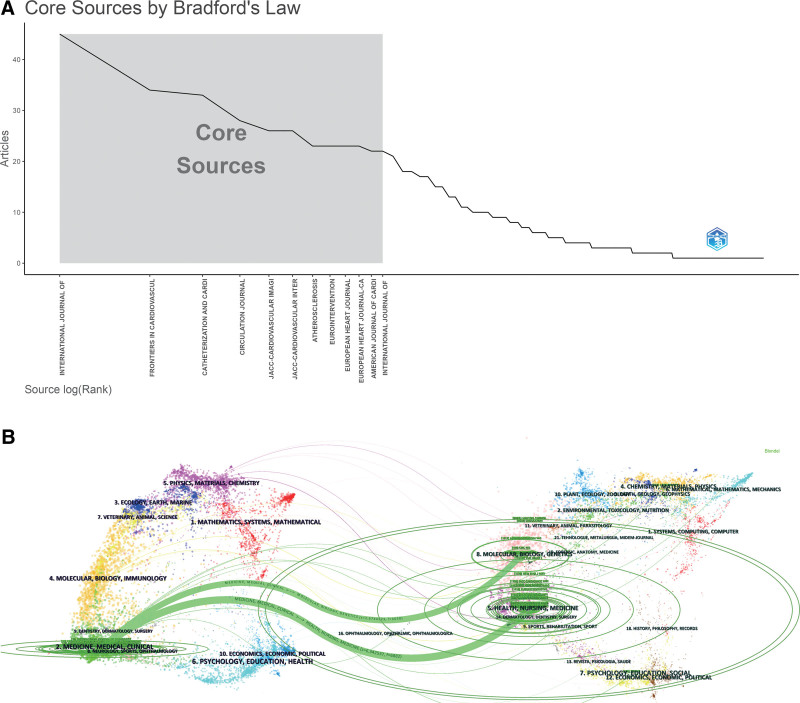
Analysis of clinical research on OCT published in academic journals. (A) Bradford’s law according to academic journals. (B) Dual map overlay of journals. OCT = optical coherence tomography.

A total of 225 journals have published research on the use of OCT in patients with ACS. Table 3 and Figure 5A show that the top 10 most productive journals published 281 papers on OCT, accounting for 29.12% of the 965 publications. The International Journal of Cardiology was the most productive journal with 45 publications on OCT. According to the JCR 2022 standards, most of the top 10 active journals were classified as Q1 or Q2.

**Table 3:** The top 10 journals.

Rank	Journal	Publications	Impact Factor™ (2022)	Journal Citation Reports ™ 2022	Citations
1	International journal of cardiology	45	3.5	Q2	711
2	Frontiers in cardiovascular medicine	34	3.6	Q2	125
3	Catheterization and cardiovascular interventions	33	2.3	Q3	328
4	Circulation journal	28	3.3	Q3	657
5	JACC-cardiovascular imaging	26	14.0	Q1	1411
6	JAC-cardiovascular interventions	25	11.3	Q1	1185
7	Eurointervention	24	6.2	Q1	813
8	European heart journal	23	39.3	Q1	8296
9	European heart journal-cardiovascular imaging	22	6.2	Q1	704
10	American journal of cardiology	21	2.8	Q3	577

This dual map provides an understanding of the past direction of development in the field of OCT in ACS. The labels on the left of the dual map represent citing journals and the labels on the right represent cited journals. A colored curve of citation connections originating from the citing map and pointing to the cited map indicates the context of the citation.^[5]^ Overall, Figure 5B shows that citing publications were mostly published in medicine/medicine/clinical medicine and molecular/biology/immunology journals, whereas most cited publications were published in health/nursing/medicine and molecular/biology/genetics journals.

### 3.4. Analysis of keywords

**Figure 6.**
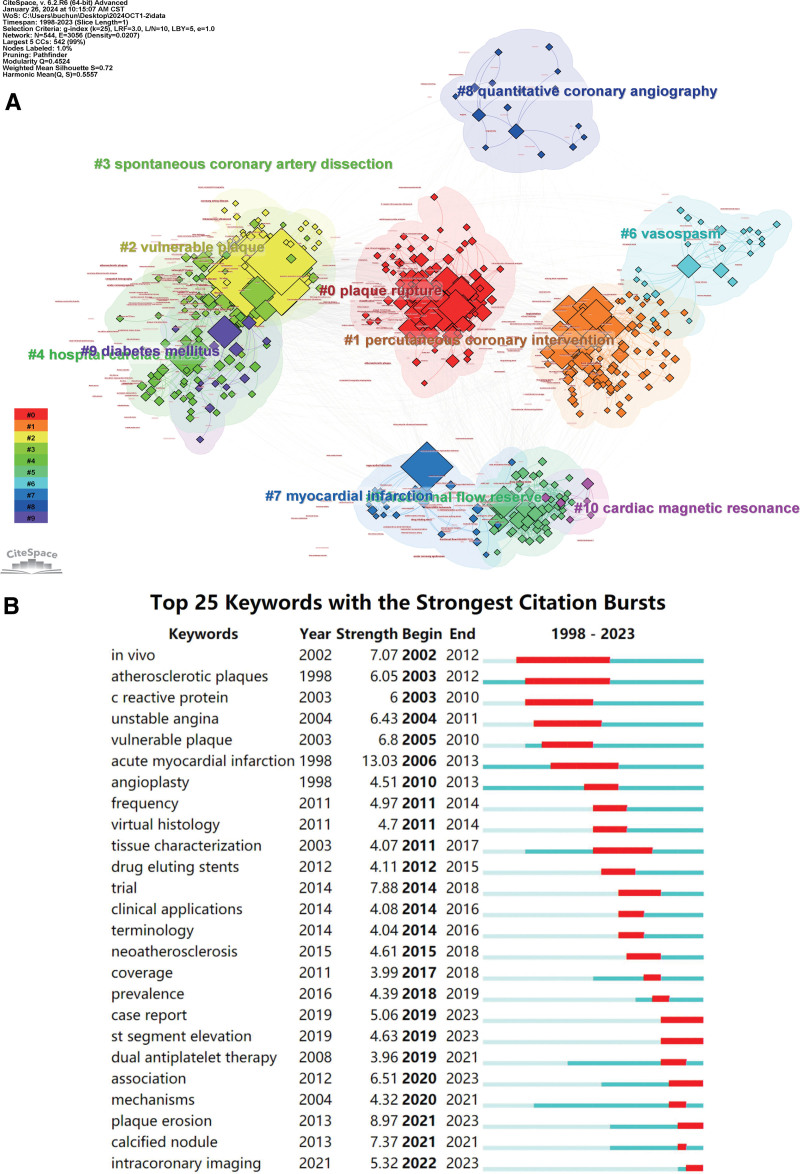
Analysis of keywords related to OCT clinical research in CiteSpace. (A) Cluster analysis of keywords. (B) The 25 keywords with the strongest citation bursts. OCT = optical coherence tomography.

Keywords reflect hotspots and frontiers in a particular field.^[6]^ CiteSpace was used to construct a keyword co-occurrence map (Fig. 6A). Of the 2547 keywords, the top 10 keywords used more than 110 times were as follows: optical coherence tomography (737 records), acute coronary syndrome (494 records), intravascular ultrasound (331 records), acute myocardial infarction (321 records), atherosclerosis (168 records), plaque rupture (128 records), thrombosis (129 records), percutaneous coronary intervention (119 records), vulnerable plaque (110 records), and coronary artery disease (109 records).

Bursts indicate that a keyword has received significant attention over time. We visualized 25 keywords with the strongest bursts (Fig. 6B) and found that “acute myocardial infarction” was the keyword with the highest burst intensity, reflecting a high-intensity outbreak from 2006 to 2013. Notably, “calcified nodul,” “plaque erosion,” and “intracoronary imaging” have been the most active burst keywords since 2021.

### 3.5. Analysis of highly cocited references

**Figure 7.**
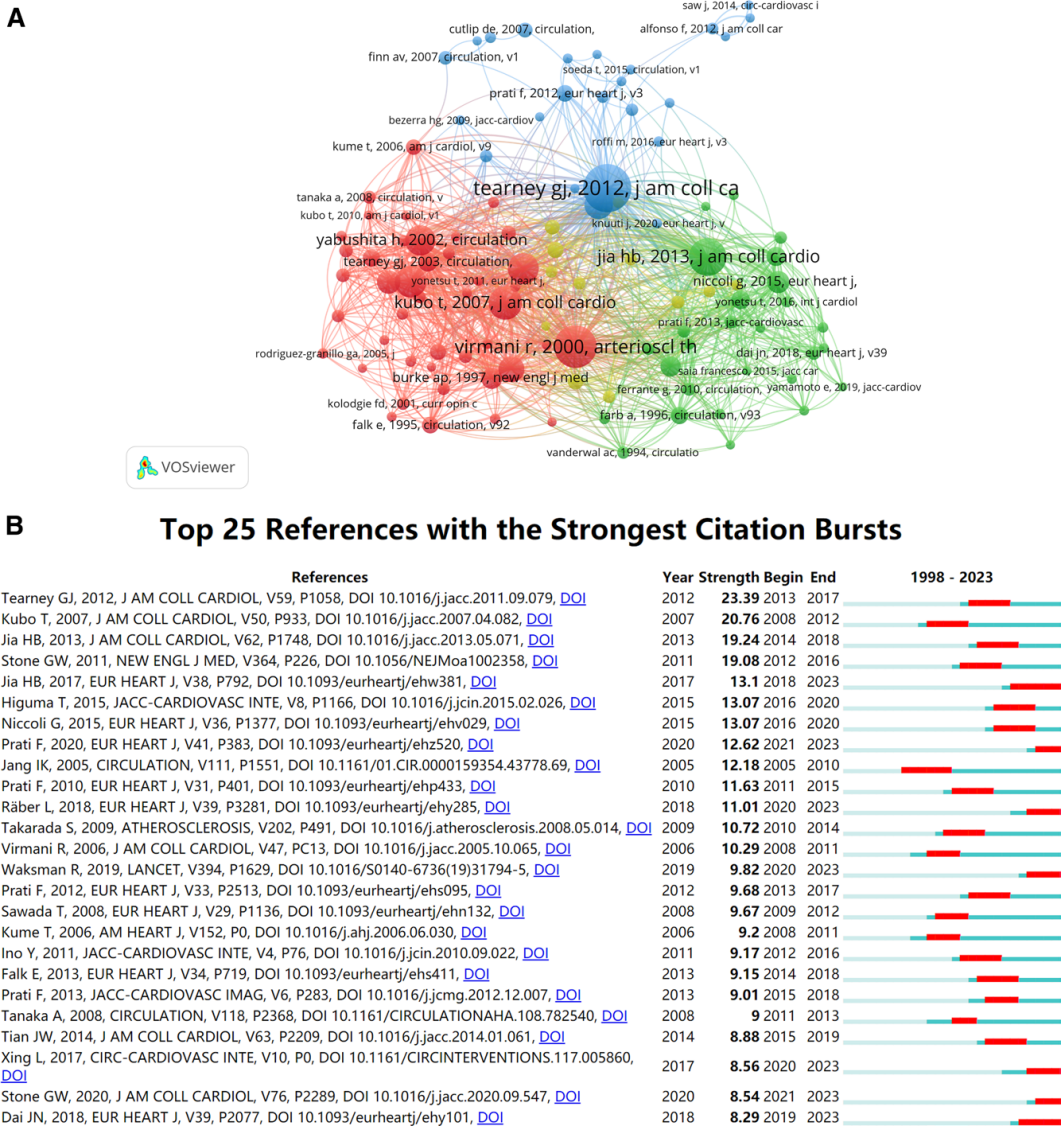
Analysis of references related to OCT clinical research. (A) Visualisation of a clustering map of highly cited references in VOSviewer. (B) The 25 most cited references with the strongest citation bursts. OCT = optical coherence tomography.

The citation of a journal refers to 2 papers cited together in another paper. In this study, we used VOSviewer to identify highly cited references, and the results showed that 20,164 references were cited. The minimum number of citations of cited references was set to 30 leaves, 106 studies, and the final network graph is shown in Figure 7A. The network graph of highly cited references can be divided into 4 clusters corresponding to the 4 colors in the graph. The red cluster is mainly associated with the morphology of the culprit lesion in patients with sudden coronary death or AMI, according to OCT (Virmani et al 2000, Kubo et al 2007). Studies in the green cluster were more likely to show plaque erosion or calcified nodules on OCT in patients with ACS (Jia et al 2013). Most studies in the blue cluster involved standardization and validation of OCT applications (Tearney et al 2012). The yellow cluster shows the relationship between high-risk OCT plaque characteristics and clinical outcomes (Kato et al 2012, Francesco Prati et al 2020). The top 10 studies with the most cited articles are shown in Table 4, including 2 reviews and 8 research articles.^[7–16]^ All of these highly cited references were published in influential and prestigious journals, such as the European Heart Journal, the Journal of the American College of Cardiology, and the New England Journal of Medicine.

**Table 4:** The top 10 highly cited references.

Rank	Article Title	Journal	Authors	Publication year	Total citation	Type of documents
1	Consensus standards for acquisition, measurement, and reporting of intravascular optical coherence tomography studies: a report from the International Working Group for Intravascular Optical Coherence Tomography Standardization and Validation	Journal of the American College of Cardiology .	Tearney GJ, et al	2012	99	Article
2	Effective anti-thrombotic therapy without stenting: intravascular optical coherence tomography-based management in plaque erosion (the EROSION study)	European Heart Journal	Jia HB, et al	2017	73	Article
3	In vivo diagnosis of plaque erosion and calcified nodule in patients with acute coronary syndrome by intravascular optical coherence tomography	Journal of the American College of Cardiology	Jia HB, et al	2013	66	Article
4	A prospective natural-history study of coronary atherosclerosis	New England Journal of Medicine	Stone GW, et al	2011	60	Article
5	Acute myocardial infarction with no obstructive coronary atherosclerosis: mechanisms and management	European Heart Journal	Niccoli G, et al	2015	52	Review
6	In vivo predictors of plaque erosion in patients with ST-segment elevation myocardial infarction: a clinical, angiographical, and intravascular optical coherence tomography study	European Heart Journal	Dai JN, et al	2018	50	Article
7	Relationship between coronary plaque morphology of the left anterior descending artery and 12 months clinical outcome: the CLIMA study	European Heart Journal	Prati F, et al	2020	48	Article
8	Assessment of culprit lesion morphology in acute myocardial infarction: ability of optical coherence tomography compared with intravascular ultrasound and coronary angioscopy	Journal of the American College of Cardiology	Kubo T, et al	2007	47	Article
9	Expert review document on methodology, terminology, and clinical applications of optical coherence tomography: physical principles, methodology of image acquisition, and clinical application for assessment of coronary arteries and atherosclerosis	European Heart Journal	Prati F, et al	2010	43	Review
10	Clinical use of intracoronary imaging. Part 1: guidance and optimization of coronary interventions. An expert consensus document of the European Association of Percutaneous Cardiovascular Interventions.	European Heart Journal	Räber L, et al	2018	42	Article

The reference bursts provide evidence that specific publications are associated with an increase in citations, suggesting that relevant literature has received increased attention in the field. Figure 7B lists the top 25 references in terms of citation bursts during the development of OCT for ACS research over the last 2 decades, reflecting the dynamics of the research field. It is noteworthy that most articles were published in high-quality or even top-tier academic journals, which may be one of the reasons why these articles were relatively hot. It is worth noting that 7 studies are still hot in 2023. Tearney et al (2012) studied publications in the Journal of the American College of Cardiology,^[7]^ which had the highest burstiness (strength = 23.39).

## 4. Discussion

This bibliometric study aimed to analyze the current status and research trends based on 1295 OCT-related ACS articles from 1389 institutions with 12,356 authors in the WoS core collection database from January 01, 1998, to December 31, 2023. These results provide insight into recent developments in published clinical research on the use of OCT in ACS. The analysis of the publication output showed that the study period could be divided into 2 phases. In the first phase, from 1998 to 2006, only to 1 to 3 articles were published each year. In the second phase, from 2007 to 2023, clinical research publications remained high, with a small peak in 2021. These results suggest that OCT-guided diagnosis and therapy of patients with ACS are attracting increasing attention.

According to the journal analysis, the top 10 most active journals published less than half (29.12%) of the research in the field of OCT use in patients with ACS. These results suggest that publications are widely dispersed across journals. The International Journal of Cardiology has been a strong publisher in recent years, showing strong interest in OCT-related research. Among the top 10 journals, 3 had an IF > 10 and > 1000 citations, indicating that some high-quality clinical trials have been conducted in this area.

In terms of national and regional distribution, the United States, Japan, and China have made greater contributions to the study of OCT in patients with ACS over the past 2 decades, as reflected in the number of publications and the frequency of citations. Among the top 10 institutions by the number of highly cited articles, 6 were from the US, with Harvard University in the first place. Although China also has an institution that ranks second in terms of OCT publications and has 4 citations, there is still much research space compared to institutions in the US. Therefore, Chinese institutions and researchers still need to improve the quality of their publications and strengthen cooperation between countries to increase their impact.

From the author’s point of view, the scientists led by Jang Ik-Kyung, Yu Bo, and Lee Hang are not only the backbone of OCT clinical research but are also highly respected in the scientific community. Their extensive research contributions have resulted in a significant number of publications and received a high citation frequency, indicating the impact and relevance of their work. Their expertise and dedication have advanced the field of OCT clinical research, paving the way for advances in diagnosis and treatment of various medical conditions.

Keyword co-occurrence analysis facilitates the estimation of hotspots and the direction of future development in a research field.^[5]^ Combined with the top 10 high-frequency keywords, the results of the keyword co-occurrence analysis can be further summarized as the following 4 hot topics: algorithms for clinicians to assess, diagnose, and treat ACS (keywords: in-hospital cardiac arrest, myocardial infarction, percutaneous coronary intervention); pathogenesis of ACS (keywords: plaque rupture, vulnerable plaque, spontaneous coronary dissection, vasospasm); risk factors analyzed in ACS populations (keywords: diabetes mellitus); and cardiac imaging (keywords: fractional flow reserve, quantitative coronary angiography, cardiac magnetic resonance). In addition, 25 keywords with the strongest citation bursts were associated with acute myocardial infarction. Calcified nodules, plaque erosion, and intracoronary imaging were the most recent burst keywords, suggesting future research directions.

OCT is a high-resolution intravascular imaging modality that can help define the nature of culprit and non-culprit lesions in patients with ACS, guide the choice of treatment strategy, and optimize the entire percutaneous coronary intervention process.^[17]^ The nature of culprit lesions in ACS that can be identified by OCT mainly include plaque rupture, plaque erosion, and calcified nodules.^[18]^ OCT is the only tool available to diagnose plaque erosion in vivo using exclusionary methods. Plaque erosion is defined as an intact fibrous cap with thrombosis and a clearly visible plaque structure under the thrombus.^[19]^ Plaque erosion can be detected in one-third of ACS cases, usually presenting as NSTEMI. In selected cases, it can be treated conservatively with dual antiplatelet therapy (DAPT), and stenting can be deferred.^[20]^ Calcified nodules are less common in patients with ACS, presenting as fibrous cap breaks on the surface of calcified plaques with thrombosis, the main features of which are thrombus adherence on the surface of the plaque and nodular calcifications projecting into the lumen or superficial calcifications.^[21]^ Patients with ACS due to calcified nodules are at an increased risk of ACS recurrence and target lesion revascularization (TLR), mainly due to the growing and protruding calcified masses.^[22]^

The analysis of cited articles and literature clusters reflects the core content and hot topics in a field. Highly cited documents may stimulate core research, while high-intensity bursts may represent emerging academic hotspots.^[23]^ The 3 most cited articles are as follows: In 2012, Tearney et al (99 citations)^[7]^ also had the highest burst intensity. This document focuses on the standardization and validation of OCT use in clinics on behalf of the OCT International Working Group. Jia HB et al 2017 (73 citations).^[8]^ The aim of this prospective study was to evaluate the feasibility and safety of antithrombotic therapy without stenting in patients with ACS with plaque erosion diagnosed using OCT. Jia et al in 2013 (66 citations) study^[9]^ characterized the morphological features of plaque erosion and calcified nodules in patients with ACS using OCT.

## 5. Limitations

This study has several limitations. First, the data were retrieved from a single WoS core collection database and did not include other databases. Second, this study only included research studies and reviews, and all the included studies were written in English. Therefore, these findings may not fully reflect the global situation in this region.

## 6. Conclusions

This bibliometric study has shown that research on the use of OCT in ACS is extensive and has developed rapidly. The United States has been the most prolific country, but Japan and China have also made significant research contributions, which has been a major force in the development of the field. Jang Ik-Kyung was the leader in the field and published the most articles. We also found that most of the top 10 productive journals had high impact factors, indicating the high academic value of OCT in ACS research. Overall, our study reports the main aspects of the topic and provides directions and references for further research on the use of OCT in ACS.

## Author contributions

**Conceptualization:** BuChun Zhang.

**Data curation:** Yi Zhang, Hui Li, Nan Chen.

**Formal analysis:** BuChun Zhang, Hui Li, Nan Chen.

**Funding acquisition:** BuChun Zhang.

**Investigation:** Yi Zhang, Hui Li.

## References

[1] Gomes PM Almeida BO Marinelli Pedrini S. Morphology and phenotype characteristics of atherosclerotic plaque in patients with acute coronary syndrome: contemporary optical coherence tomography findings. Coron Artery Dis. 2021;32:698–705. 33587362 10.1097/MCA.0000000000001027

[2] Cho JM Sohn IS Jang IK. Late sequela of Kawasaki’s disease: Optical coherence tomographic finding. J Invasive Cardiol. 2009;21:668. 19966372

[3] Karimi Galougahi K Dakroub A Chau K. Utility of optical coherence tomography in acute coronary syndromes. Catheter Cardiovasc Interv. 2023;102:46–55. 37245076 10.1002/ccd.30656

[4] Ninkov A Frank JR Maggio LA. Bibliometrics: Methods for studying academic publishing. Perspect Med Educ. 2022;11:173–6. 34914027 10.1007/s40037-021-00695-4 PMC9240160

[5] Chen C Leydesdorf L. Patterns of connections and movements in dualmap overlays: a new method of publication portfolio analysis. J Assoc Inf Sci Technol. 2014;65:334–51.

[6] Zhou Q Kong HB He BM Zhou SY. Bibliometric analysis of bronchopulmonary dysplasia in extremely premature infants in the web of science database using CiteSpace software. Front Pediatr. 2021;9:705033. 34490163 10.3389/fped.2021.705033 PMC8417835

[7] Tearney GJ Regar E Akasaka T.; International Working Group for Intravascular Optical Coherence Tomography (IWG-IVOCT). Consensus standards for acquisition, measurement, and reporting of intravascular optical coherence tomography studies: a report from the International Working Group for Intravascular Optical Coherence Tomography Standardization and Validation. J Am Coll Cardiol. 2012;59:1058–72. 22421299 10.1016/j.jacc.2011.09.079

[8] Jia H Dai J Hou J. Effective antithrombotic therapy without stenting: intravascular optical coherence tomography-based management in plaque erosion (the EROSION study). Eur Heart J. 2017;38:792–800. 27578806 10.1093/eurheartj/ehw381

[9] Jia H Abtahian F Aguirre AD. In vivo diagnosis of plaque erosion and calcified nodule in patients with acute coronary syndrome by intravascular optical coherence tomography. J Am Coll Cardiol. 2013;62:1748–58. 23810884 10.1016/j.jacc.2013.05.071 PMC3874870

[10] Stone GW Maehara A Lansky AJ.; PROSPECT Investigators. A prospective natural-history study of coronary atherosclerosis. N Engl J Med. 2011;364:226–35. 21247313 10.1056/NEJMoa1002358

[11] Niccoli G Scalone G Crea F. Acute myocardial infarction with no obstructive coronary atherosclerosis: mechanisms and management. Eur Heart J. 2015;36:475–81. 25526726 10.1093/eurheartj/ehu469

[12] Dai J Xing L Jia H. In vivo predictors of plaque erosion in patients with ST-segment elevation myocardial infarction: a clinical, angiographical, and intravascular optical coherence study. Eur Heart J. 2018;39:2077–85. 29547992 10.1093/eurheartj/ehy101

[13] Prati F Romagnoli E Gatto L. Relationship between coronary plaque morphology in the left anterior descending artery and 12-month clinical outcome: the CLIMA study. Eur Heart J. 2020;41:383–91. 31504405 10.1093/eurheartj/ehz520

[14] Kubo T Imanishi T Takarada S. Assessment of culprit lesion morphology in acute myocardial infarction: ability of optical coherence tomography compared with intravascular ultrasound and coronary angioscopy. J Am Coll Cardiol. 2007;50:933–9. 17765119 10.1016/j.jacc.2007.04.082

[15] Prati F Regar E Mintz GS.; Expert's OCT Review Document. Expert’s OCT review document. Eur Heart J. 2010;31:401–15. 19892716 10.1093/eurheartj/ehp433

[16] Räber L Mintz GS Koskinas KC.; ESC Scientific Document Group. Clinical use of intracoronary imaging. Part 1: guidance and optimization of coronary interventions. An expert consensus document of the European Association of Percutaneous Cardiovascular Interventions. Eur Heart J. 2018;39:3281–300. 29790954 10.1093/eurheartj/ehy285

[17] Harman J. Unravelling ACS pathophysiology using intracoronary OCT and deep immunophenotyping. Nat Rev Cardiol. 2023;20:581. 10.1038/s41569-023-00915-w 37491589

[18] Ahn JH Kim MC Ahn Y. Culprit lesion plaque characteristics and angiopoietin like 4 in acute coronary syndrome: A virtual histology-intravascular ultrasound analysis. Int J Cardiol. 2023;388:131164. 37429444 10.1016/j.ijcard.2023.131164

[19] Fahed AC Jang IK. Plaque erosion and acute coronary syndromes: phenotype, molecular characteristics and future directions. Nat Rev Cardiol. 2021;18:724–34. 33953381 10.1038/s41569-021-00542-3

[20] Collet C Conte E Mushtaq S. Reviewing imaging modalities for the assessment of plaque erosion. Atherosclerosis. 2021;318:52–9. 33129585 10.1016/j.atherosclerosis.2020.10.017

[21] Sugiura J Watanabe M Nobuta S. Prediction of optical coherence tomography-detected calcified nodules using coronary computed tomography angiography. Sci Rep. 2022;12:22296. 36566340 10.1038/s41598-022-26599-9 PMC9789942

[22] Lei F Yin Y Liu X. Clinical outcomes of different calcified culprit plaques in patients with acute coronary syndrome. J Clin Med. 2022;11:4018. 35887782 10.3390/jcm11144018 PMC9316434

[23] Tuerdi R Zhang H Wang W Shen M Wei X. Bibliometric analysis of the research hotspots and trends of circular RNAs. Heliyon. 2024;10:e31478. 38818139 10.1016/j.heliyon.2024.e31478 PMC11137546

